# A Deep Learning Approach to the Screening of Oncogenic Gene Fusions in Humans

**DOI:** 10.3390/ijms20071645

**Published:** 2019-04-02

**Authors:** Marta Lovino, Gianvito Urgese, Enrico Macii, Santa Di Cataldo, Elisa Ficarra

**Affiliations:** 1 Politecnico di Torino, Department of Control and Computer Engineering, Corso Duca Degli Abruzzi 24, 10129 Torino, Italy; gianvito.urgese@polito.it (G.U.); santa.dicataldo@polito.it (S.D.C.); elisa.ficarra@polito.it (E.F.); 2 Politecnico di Torino, Interuniversity Department of Regional and Urban Studies and Planning, Corso Duca Degli Abruzzi 24, 10129 Torino, Italy; enrico.macii@polito.it

**Keywords:** machine learning, deep learning, convolutional neural networks (CNN), gene fusions, protein function, gene fusion detection tools, oncogenic probability value

## Abstract

Gene fusions have a very important role in the study of cancer development. In this regard, predicting the probability of protein fusion transcripts of developing into a cancer is a very challenging and yet not fully explored research problem. To this date, all the available approaches in literature try to explain the oncogenic potential of gene fusions based on protein domain analysis, that is cancer-specific and not easy to adapt to newly developed information. In our work, we choose the raw protein sequences as the input baseline, and propose the use of deep learning, and more specifically Convolutional Neural Networks, to infer the oncogenity probability score of gene fusion transcripts and to group them into a number of categories (e.g., oncogenic/not oncogenic). This is an inherently flexible methodology that, unlike previous approaches, can be re-trained with very less efforts on newly available data (for example, from a different cancer). Based on experimental results on a large dataset of pre-annotated gene fusions, our method is able to predict the oncogenity potential of gene fusion transcripts with accuracy of about 72%, which increases to 86% if we consider the only instances that are classified with a high confidence level.

## 1. Introduction

Nowadays, the increased availability of Next Generation Sequencing (NGS) data enables new unforeseen insights into the relation between some genetic rearrangements and cancer development. In this regard, a challenging area is represented by the study of gene fusions, a genetic aberration where two separate DNA regions (usually two distinct genes) join together into a hybrid gene. The genes retained at 5p’ and 3p’ of the fused sequence are conventionally called 5p’ gene and 3p’ gene, respectively. If the promoter region of at least one of the two genes is retained in the fusion, the erroneous sequence is transcribed at the RNA level, and the aberrated transcript can result into an abnormal protein [1].

Since the discovery of the first genetic rearrangement by Nowell and Hungerford in 1960, a large number of gene fusions have been associated with cancer development and used as cancer predictors [1]. However, gene fusions do not automatically relate to oncogenic processes, as they can be found in large number even in non-tumoral samples [2]. In light of the above, predicting whether an aberrated transcript will result into a protein with an oncogenic effect or not is a very critical and challenging task in the study of cancer development.

Traditionally, many methodologies have been used for the identification of fusion genes (e.g., fluorescence in situ hybridization (FISH) [3] or comparative genomic hybridization (CGH) [4]). In recent years, the spreading of NGS technologies has enabled the development of gene fusion detection tools, whose aim is to identify chimeric transcripts exploiting information coming from RNA paired-end sequencing data [5].

Typically, the analysis of such data consists of three main phases:

1. primary identification of candidate gene fusions.
2. filtering of the fusion candidates, based on the number of reads mapping to a specific region and/or on the functional annotation of the involved genes. The outcome of this phase is a sub-set of candidates with best read quality mappings and/or highest probability of resulting into a functional oncogenic product.
3. in situ validation of the fusions resulting from phase 2.

The first phase of the analysis is performed using fusion detection tools (among the others, Chimerascan [6], Defuse [7], Prada [8] and many more [9,10,11]). To date, the major issue with the outcome of these tools is related to the interpretation of the chimeric transcripts that are found. Given the high validation costs of each gene fusion, extensive post-processing efforts are devoted into distinguishing driver fusions from passenger mutations, in order to reduce the number of false positives in the last part of the pipeline. This makes the second phase of the analysis particularly critical and challenging.

While the majority of the tools in literature apply filtering criteria based on read mapping quality (among the others, Tophat-fusion [12] and Star-fusion [13]), a complementary approach for the interpretation of gene fusion candidates consists in a functional study of the chimeric transcript, looking at possible similarities with cancer genes: the higher the similarity, the higher the probability of developing into cancer. This similarity analysis involves specific functional annotations, protein interactions as well as protein domain analysis [14].

To perform a full functional study of a chimeric transcript, all of the available approaches in literature reconstruct the candidate fusions and then apply different types of machine learning methods to perform protein domain analysis [15,16]. Given the uncertainty on the training set, these tools mainly use predictive models to derive conserved and lost protein domains in fusions, and then exploit the outcome of such predictions to train a machine learning method. The most popular tool in this category is Oncofuse [15], which assigns a functional prediction score (oncogenic potential, i.e., the probability of being driver events) to the fusion sequences exploiting a naive Bayesian classifier.

While information on conserved or lost protein domains is generally successful to prioritize on the candidate fusions, a well-known drawback of this approach is its lack of flexibility, in that any change in the classification problem (either a different type of cancer, or newly acquired information) requires significant efforts devoted into re-parametrization of the model and laborious re-derivation of the protein domains. This is a very inconvenient trait, especially if we consider that the study of cancer development is built on top of continuously evolving information.

In this work, we continue on the path of the functional annotation of the chimeric transcript (phase 2 of the analysis pipeline), but with a more flexible approach. We exploit human reference sequences, relying only on the raw fusion sequence information, with no additional input about conserved or lost protein domains. By doing so, our aim is to avoid any possible bias that the prediction models leveraged by protein domain analysis may introduce into the classification task, as well as to improve the generalization capabilities and ease-of-retraining of the classifier.

The proposed solution is based on Convolutional Neural Networks (CNNs), a class of deep, feed-forward neural networks with the inbuilt ability of automatically learning the most significant classification features directly from the raw input data [17,18]. Hence, they avoid the necessity of designing handcrafted descriptors, which may be difficult to generalize to different classification problems. Thanks to this peculiar characteristics, they can easily adapt to newly acquired information, by simply re-running the automated back-propagation algorithm on the new training data. Originally designed for image classification tasks, CNNs are now successfully applied to most pattern recognition and classification problems, from computer vision [19] and natural language processing [20] to bioinformatics (for example, to the prediction of single-cell DNA methylation states and microRNA targets, as well as to the recognition of splice junction sites and promoter sequence regions [21]).

To design a model that is completely independent from protein domain information, in our work, we feed a CNN directly with the the real amino-acid composition of the fused proteins, with no additional data interpretation. The output of the network consists in a 0–1 score, which can be interpreted in terms of probability of the input chimeric transcript of being involved in an oncogenic process. This score can also be translated into a categorical class label, partitioning the input gene fusions into two different groups (oncogenic or not oncogenic, respectively), with a corresponding confidence level.

The structure of this paper is the following. In Section 2, we describe and discuss our experimental results. In Section 3, we describe in detail our proposed solution, providing the design and implementation details of the CNN. Finally, in Section 4, we provide our final remarks and future work.

## 2. Results and Discussion

Summarizing, the purpose of our methodology is two-fold: (i) to automatically discriminate between gene fusions with functional oncogenic potential (referred to as Onco class) and fusions that are not involved in an oncogenic process (referred to as NotOnco class), without any additional information on the protein domains retained or lost in the fusion sequences; (ii) to provide, along with the class category each fusion sequence belongs to, a score representing the probability of that sequence being oncogenic. For this purpose, we exploit the inbuilt ability of the CNN of recognizing local spatial patterns that are significant for the classification directly from the input sequences, without requiring any a priori description of the most relevant features of the two classes.

Overall, our dataset contains a total number of 2318 reconstructed fused sequences, respectively 1158 for the Onco class and 1160 for the NotOnco class. To solve our classification problem, we randomly partitioned this dataset into a training and a test set (containing 1854 and 464 samples, respectively), ensuring complete independence of the two datasets not only in terms of sequences, but also of genes involved in the fusions. Hence, the genes that are involved in fusions of the training set are not involved in the ones of the test set, and vice versa.

CNNs were originally designed for image classification tasks, hence they take numerical matrices as input. To adapt the CNN to our specific classification problem, we applied a one-hot encoding method, transforming the fusion sequences into matrix data structures that can be directly fed into the CNN. After careful configuration and tuning of the network architecture, we designed a CNN model with the following architecture: two convolutional layers of kernel size 10 (each followed by a pooling layer of kernel size 5 and by dropout regularizer set to 0.1) and two dense layers after flattening operation. The complete process of data retrieval, one-hot encoding from sequence to data matrix and CNN design and training is described in details in Section 3.

### 2.1. Fusion Annotation Performance

Following the two-fold purpose of our work, given a new protein sequence resulting from a gene fusion event, our proposed CNN returns: (i) a categorical label assigning the input sequence to either the Onco or the NotOnco class; and (ii) a real value, representing the probability of the input sequence being oncogenic. This probability value is a real number in a (0,1) range, where 0 means definitely not oncogenic and 1 definitely oncogenic. The binary class label and the probability score are strictly related to each other, in that former is obtained by setting a threshold on the latter, as follows:

(1) $$Class\;label=\left\{\begin{array}{cc} NotOnco & \mathrm{if}\;prob.\;value<0.5,\\ Onco & \mathrm{if}\;prob.\;value\geq 0.5. \end{array}\right.$$

Hence, the probability score can be even interpreted in terms of binary classification confidence: the closer the probability score to 0, the higher the confidence of the class NotOnco and, vice versa, the closer to 1, the higher the confidence of the class Onco.

The performance of our CNN model was first assessed in terms of binary classification accuracy (i.e., accuracy in the discrimination between Onco and NotOnco classes. For this purpose, we run the trained model on the test set, which contains an almost equal number of protein sequences of the two classes. The obtained results are represented in the confusion matrix of Table 1, with rows reporting the number of instances classified in the two class categories, and columns reporting the number of instances in the actual classes, respectively. Hence, the values in the main diagonal represent correct classifications, and values outside the main diagonal the misclassifications.

As it can be gathered from the confusion matrix, the performance of the CNN in terms of binary annotation of the fusion sequences was quite balanced in the two classes. If we consider the overall number of instances in the test set, the classification accuracy of the CNN was 71.55%, with precision and recall of the Onco class equal to 74.50% and 65.51% respectively.

As already mentioned, along with the categorical class label, the CNN provides a (0,1) oncogenic probability value. As this value is closely related to the confidence level of the classification label, it needs to be taken into due consideration when interpreting the classification outcome. This is done in the second part of our experimental analysis.

**Figure 1:**
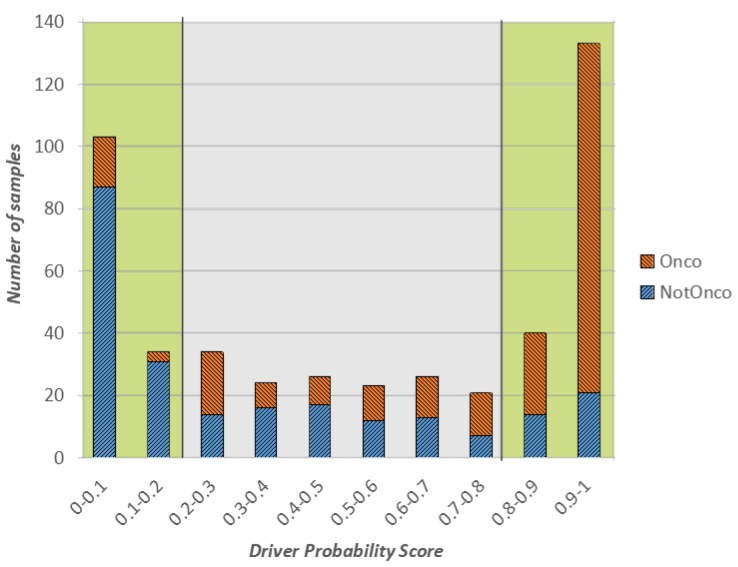
Classification outcome on the test set: class label vs. oncogenic probability score.

In Figure 1, we show a stacked plot of the outcome of the CNN on the test set, grouped by the oncogenic probability value predicted by the model. More specifically, the *x*-axis represents the oncogenic probability values divided into 10 ranges of equal width. Each bar in the plot reports the number of test instances (in orange the ones whose actual class is Onco and in blue the ones whose actual class is NotOnco) that obtained a predicted oncogenic probability in a certain range. For example, if we consider the first bar on the left, 103 samples of the test set received an oncogenic probability value between 0 and 0.1, and following Equation (1) they were labelled as NotOnco by the model. Hence, the height of the bar is 103. Of these 103 samples, 87 actually belonged to the NotOnco, represented by the blue color, and remaining 16 to the NotOnco class, represented in orange. Then, the higher the proportion of the blue coloring in the bars on the left side of the plot, the better the classification outcome of the NotOnco class. Likewise, the higher the proportion of orange coloring in the bars on the right side, the better the classification outcome of the Onco class.

When associated with the binary class label, the oncogenic probability value can be interpreted as a level of confidence of such label: the lower the value, the higher the confidence of the NotOnco class, the higher the value, the higher the confidence of the Onco class. To represent this concept, the extreme left and right areas of the plot (the ones with oncogenic probability lower than 0.2 and higher than 0.8, respectively), have a green-colored background. Instances in these two areas are the ones that were classified with highest confidence by the CNN. Likewise, the instances with oncogenic probability between 0.2 and 0.8 are the ones for which the CNN outcome is most uncertain, represented by a grey-colored background.

Overall, as it can be easily seen from Figure 1, most of the classification outcomes of our proposed solution are located in the green regions, either close to 0 (surely not oncogenic) or close to 1 (surely oncogenic). More specifically, more than 65% of test set samples were classified by the CNN with confidence higher than 80%, which means that the corresponding class label can be considered reliable.

If we consider the only instances in the the green areas of the plot, the classification accuracy was very high, respectively, 86% for Onco and 80% for NotOnco. Hence, the Onco class was classified with a precision of 86% and recall of 89%. Most of the misclassifications occurred in the gray areas of the plot, where there is not a clear prevalence of either orange or blue in the bars. As the classification label and the oncogenic probability score should be considered jointly, the user may decide to overlook the classification outcome on the gene fusions of the low-confidence area.

### 2.2. Comparison with the Outcome of Oncofuse and Pegasus

The proposed model and the traditional machine learning tools for gene fusion annotation (e.g., Oncofuse [15] and Pegasus [16]) rely on completely different approaches. While our solution uses the fusion sequence as the only information to distinguish between oncogenic and not oncogenic fusions, the other tools apply predictive models exploiting conserved and lost protein domains information to infer the oncogenic potential of gene fusions. Hence, while our approach can automatically adapt to miscellaneous protein features by simply re-running the backpropagation algorithm on a new set of gene fusions, the traditional tools do not allow the same level of flexibility. On this premise, to show the potentials of CNN model from the point of view of a regular user in a real world setting, we compare the outcome of our CNN approach with the outcome of OncoFuse and Pegasus on the same input data (i.e., the only fusion transcript sequences).

The same as our approach, both Oncofuse and Pegasus assign a (0,1) oncogenic probability score to the fusion sequences, which can be exploited to evaluate strength points of our proposed solution.

**Figure 2:**
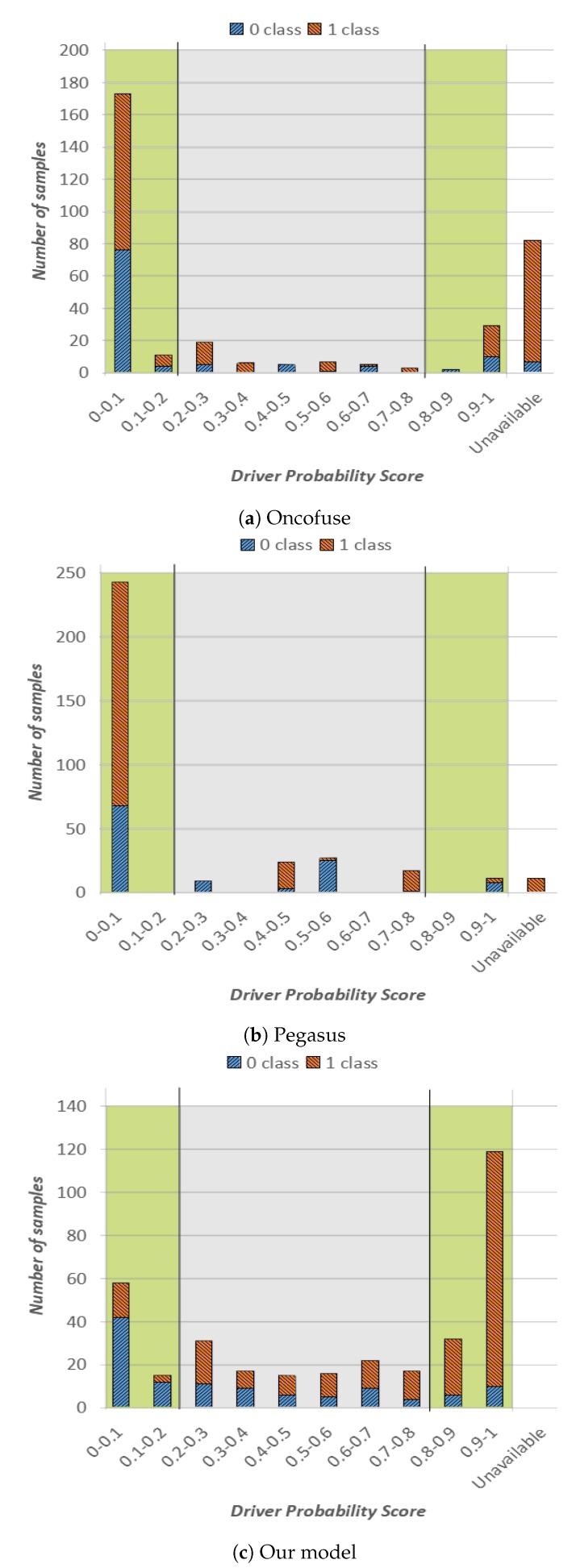
Classification outcome on the test set for Oncofuse, Pegasus and our proposed model.

In our experiments, we run Oncofuse, Pegasus and our tool on the same set of gene fusion sequences (i.e., the ones of the test set), properly formatting data according to the input required by each tool. Based on our experiments, OncoFuse and Pegasus tools were able to provide an outcome for 76% and 97% of the available gene fusions in our test set, respectively. The complete overview of our experiments is shown in Figure 2. More specifically, in Figure 2a, we show the outcome of Oncofuse, in Figure 2b the outcome of Pegasus and in Figure 2c the outcome of our method. The representation format is the same as Figure 1, except for the last column on the right, which reports sequences for which the corresponding tool was not able to provide a driver probability score.

From the analysis of Figure 2, we can draw the following considerations. Oncofuse had most of the classification outcomes in the green high-confidence area, and more specifically in the one of the NotOnco class (Figure 2a). As it can be easily seen in Figure 2b, Pegasus followed the same trend, except for a small number of samples in the central area, characterized by driver probability score in range (0.4–0.6). Hence, for both tools, there is a very clear prevalence of instances classified as NotOnco (i.e., 82% and 83%, respectively, for OncoFuse and Pegasus). On the other hand, the higher confidence of the classification outcome does not translate into higher classification accuracy: as it can be easily seen by the plot, both the extreme-left and the extreme-right bars both for Oncofuse and Pegasus do not have a clear prevalence of the correct classification label over the other one. In addition, for both tools, the majority of the instances in the first bar are represented by misclassifications (i.e., Onco sequences incorrectly classified as NotOnco). More specifically, more than half of the instances with a driver probability score smaller than 0.1 for OncoFuse and more than 70% for Pegasus were misclassified. If we consider the whole high-confidence area, Pegasus correctly classified only 28% of the instances against 46% of OncoFuse. On the other hand, as already pointed out in the previous analysis, 84% of the high-confidence classifications provided by our method were correct classifications. Indeed, in Figure 2c, we see a clear prevalence of blue colored bars on the left side of the plot, and a clear prevalence of orange on the right side. On top of that, the output of the classifier is more balanced among NotOnco and Onco class, respectively.

Hence, if we consider jointly classification label and probability score on our test set, the proposed solution provided a more reliable classification outcome than the one provided by OncoFuse and Pegasus. A possible explanation of these results is that OncoFuse and Pegasus exploit domain analysis models that might not perfectly fit the data used for our tests. On the other hand, unlike OncoFuse and Pegasus, the most significant feature of our solution is that it is inherently flexible because it can be automatically adapted to a new dataset by simply re-running the backpropagation algorithm.

## 3. Materials and Methods

**Figure 3:**
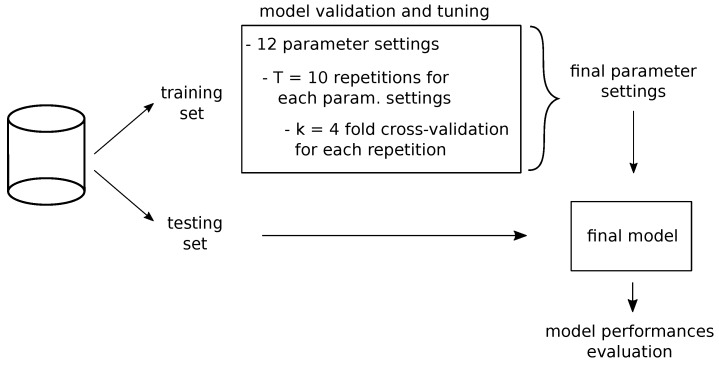
Overview of the entire process.

The design and development of our proposed solution consisted of the following main steps: (i) creation of a specific purpose dataset (with training and testing sub-sets), (ii) definition of the encoding paradigm to make the data-format usable by the CNN model, (iii) setup and tuning of the model, and (iv) model testing. The overall model design process is summarized in Figure 3 and described detail in the Section 3.1, Section 3.2, and Section 3.3.

### 3.1. Dataset

Gene fusion data were retrieved from two different sources, respectively for the Onco and the NotOnco class.

Cosmic, a catalogue for somatic mutations in cancer [22], was used for the *Onco class*. This catalogue, among the various mutations involved in oncogenic processes, in *Complete Fusion Export* section provides a list of gene fusions from solid tumors. For consistency with the NotOnco dataset, which mostly contains gene fusions from solid tumors, we selected Cosmic as the source for Onco dataset. In detail, of all gene fusion translocation names reported, we selected those for which all the information necessary to reconstruct the sequence was present (e.g., exact position of the breakpoint, the transcript names of both 5p’ and 3p’ genes and any inserted sequence across the breakpoint). From this information, we reconstructed a total of 1158 protein sequences for the Onco class. In addition, since the NotOnco class data are aligned on GRCh37, we used the GRCh37 assembly of the human genome.

Data for the NotOnco class were reconstructed based on Babicenau at al. work on recurrent chimeric fusion RNAs in non-cancer tissues and cells [2]. In this work, SOAPfuse (a tool for gene fusion analysis) was applied on 171 non-neoplastic tissue samples from 27 different tissues, identifying over 10,000 fusion events. For each of these fusions, authors report the breakpoint position on GRCh37 human reference genome of both fused genes.

In order to reconstruct the fusion proteins starting from genomic breakpoints, we considered those fusions for which both the genes involved in the fusion are protein-coding genes. In addition, all the possible isoforms of each gene were considered, discarding the non protein-coding transcripts. In this work, additional variants were not taken into account. As a matter of fact, a large number of variants consist in single nucleotide polymorphism (SNP), where bases changes do not necessarily result into a change of the final amino acids sequence and generate either duplicates or highly similar protein sequences.

To address the imbalance in the number of samples belonging to the two classes without introducing artificial examples in the dataset, we decided to down-sample the largest class (NotOnco class). In detail, to build the NotOnco class, we first selected recurrent fusions (that is, fusions present in more than one tissue or sample) and integrated them with some of the non-recurrent fusions. To avoid introducing possible biases in the classification process, we ensured a comparable distribution of protein lengths in both the classes. This configuration led to obtaining 1158 and 1160 protein sequences, respectively, for the Onco and NotOnco class. Finally, if we define a *gene pair* the pair of names of 5p’ gene and 3p’ gene, we assured that no *gene pair* is present in both Onco and NotOnco class.

In order to assess the model’s ability to recognize the oncogenic potential of new fusion proteins, we divided the overall dataset (2318 sequences) into training and test sets. In detail, to generate the test set, we selected 464 sequences (i.e., one fifth of the total), so that the *gene pairs* of the test set were not present in the training set. By doing so, we ensured a complete independence of the test set from the training set, both in terms of sequences and in terms of genes involved in the fusions.

### 3.2. Encoding: From Sequences to Matrices

Once all the fused sequences had been reconstructed, they were translated into protein sequences following the Amino-Acid Translational Table. We started the translation from the beginning of the coding sequence identifiable by the initiation codon, usually the *ATG* triplet.

As CNNs are originally designed to take images as input, the fused amino-acid sequence needs to be converted into a N×M×C data structure, where *N* and *M* represent length and width of the input image and *C* the number of channels. For our purposes, *N* was set to 5000, and shorter sequences were padded using a fake amino-acid after the stop codon triplet. By doing so, we obtained a total number of 2318 strings (464 and 1854, respectively, for test and training sets) of 22 different letters, each corresponding to one amino-acid (20 real amino-acids plus two fake ones for the stop codons and the sequence padding, respectively).

The most popular methods for encoding strings into numerical data structures are ordinal encoding and one-hot encoding, eventually with some variations. Ordinal encoding substitutes the *i*th letter in a fusion with a fixed value corresponding to a unique amino-acid. Hence, the resulting matrix will have minimal dimensions N=5000×M=1×C=1, with memory saving advantages compared to other techniques. However, the incremental values assigned to the amino-acids establish an artificial ordering of the input elements (and, hence, of the corresponding weights in the neural network) which may bias the representation and negatively affect the classification accuracy [23].

On the other hand, one-hot encoding assigns to the *i*th letter a vector of length *L*, where each *j*th element corresponds to a feature. In our work, features are the amino-acids: hence, the *i*th letter is encoded by a vector of all zeros, except for the *j*th element associated with the amino-acid, which is set to 1. This encoding procedure requires more memory compared to ordinal encoding; however, it has the strong advantage of representing the amino-acids in a unique and unbiased way.

Since optimization of memory use is not the primary goal of this work, we decided to convert the sequences into data matrices as faithfully as possible and without introducing bias into the classifier, using one hot encoding strategy. As the CNN model will inherently assume spatial correlations between adjacent matrix elements, which in our case we want to avoid, the data structure was arranged so that the amino-acid features constitute the third dimension (i.e., channels) of the matrix.

**Figure 4:**
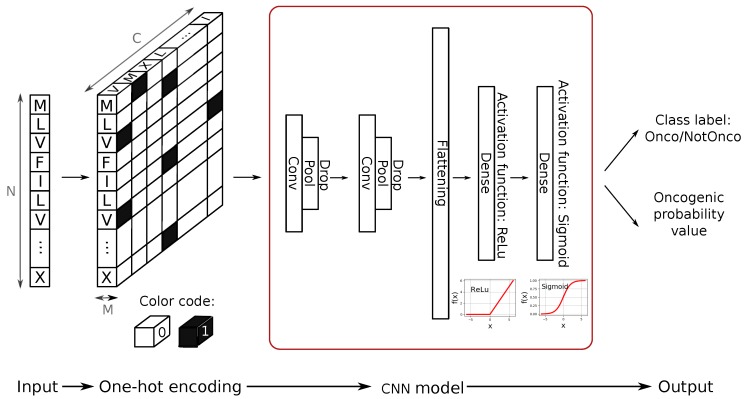
Proposed solution: from input sequence to classification outcomes (class label and oncogenic probability value).

Overall, the encoding step is summarized in the first section of Figure 4.

### 3.3. CNN Architecture, Training and Tuning Paradigm

In order to predict the oncogenic potential of proteins, we exploited the ability of CNN to learn higher-order features in the data via convolutions using two main building blocks: feature-extraction (learning) layers and classification layers [24].

As shown in the second section of Figure 4, the CNN model we designed consists of two convolutional layers (kernel size Conv) followed by two max pooling layers (kernel size Pool) that are responsible for the feature-extraction module. In order to keep the evolution of the training phase under control and to avoid overfitting, we introduced dropout regularizer (Drop parameter) and batch-normalization in our design. This technique applies a transformation that keeps the mean activation of the units close to 0.0, while also keeping the activation standard deviation close to 1.0. Batch normalization in CNNs has been demonstrated shown to significantly speed up the training, by introducing the normalization into the network architecture [24]. In the proposed model, the classification layers of the CNN first perform a flattening of the output of the feature-extraction module and then apply a 128-unit dense layer with ReLU activation function, plus a final single unit dense layer with sigmoid activation function. This final layer provides the classification output (see Figure 4).

In the training process, we set the following parameters: learning rate equal to 0.005, batch size 128 and number of epochs 50 with early stopping condition to reduce the overall training time (in our case, if there are no improvements after 30 epochs, we stop the training process). The network was trained by backpropagation, by implementing a Stochastic gradient descent optimizer [25].

The CNN was implemented in the Keras python library under Tensorflow backend [26].

In order to obtain the best architectural parameters of our model, we implemented stratified *k*-fold cross validation, splitting the training dataset into k=4 completely independent bins, so that *gene pair*s of 5p’ and 3p’ genes in each bin are not present in any of the other bins. Furthermore, we ensured in each bin an equal number of samples both for the Onco and for the NotOnco class. In addition, in order to consider the effects of random initialization of the network weights, as well as of other functions (e.g., dropout regularizer), the cross-validation was run T=10 times, obtaining 10 cross-validation accuracy values per each parameters configuration (i.e., Conv, Pool and Drop parameters). Then, to decide the best configuration, we computed median and standard deviation of these accuracy values.

As the setting parameters of a neural network are not independent from each other, in order to tune the hyper-parameters we performed a grid search over (i) Conv, (ii) Pool and (iii) Drop values, obtaining a total number of 12 possible network configurations. The obtained results per each configuration are reported in Table 2.

Based on the joint analysis of the median and standard deviation of the *T* cross-validation accuracy values, we selected Conf_10 as the final configuration setting. This is the setting that ensured the best compromise between relatively high cross-validation accuracy and a contained standard deviation over the *T* repetitions. Hence, we used this setting to train the final model on training set. Nonetheless, by looking at Table 2, we can see that the performance of the CNN was relatively stable when changing the parameters setting.

### 3.4. Samples Availability

Training and testing datasets used in this work are available from the authors on request.

## 4. Conclusions and Future Works

In this paper, we suggested that the only amino-acid sequence is enough to predict the oncogenic potential of a protein sequence resulting from a gene fusion. Based on this hypothesis, we proposed a CNN model that takes the amino-acid sequence as input, without any additional information about protein domains. This is a much more flexible approach compared to the available annotation tools, as the CNN can be easily re-adapted to different cancers or to newly acquired information by simply re-running the automated backpropagation algorithm on a new training set.

Even though the model is to date intrinsically limited by the scarcity of the training data, it achieved a good classification accuracy on the test set. By setting an adequate threshold on the prediction confidence of the model, we were able to classify more than 65% of the samples with accuracy higher than 80%, overcoming the predictions obtained by Oncofuse both in terms of classification accuracy and reliability of the prediction.

As a matter of fact, the precision of the tool in a clinical application, where the number of true NotOnco transcripts can be larger by at least an order of magnitude compared to true Onco transcripts, still has room for improvement. However, as shown by our analysis, the proposed solution has significant advantages compared to the state of the art, as it provides a flexible and effective support to the first prioritization of the gene fusions to investigate.

In fact, in the validation process of protein fusion sequences, it is important to consider not only the actual presence of the fusion in the sample, but also the functional impact of that fusion on an oncogenic process. Because of the high validation costs of the fusions, keeping the number of false positives (i.e., non-oncogenic fusions misclassified as ongenic) under control is fundamental. To reduce false positives of the fusion detection tools, the user can filter candidate fusions based on the oncogenic probability value provided by our model, retaining only the fusion sequences for which the classification label is provided with sufficiently high confidence.

Future works will focus on two main directions. The first direction is the improvement of the predictive model, with special attention to further reduction of the false positive rates. This will require a significant enlargement of the training set. The second direction is the interpretation of the features automatically extracted by the CNN model. These features can be exploited to obtain a deeper understanding of the specific biological patterns that mostly influence the oncogenic potential of a gene fusion.

## Figures and Tables

**Table 1:** Onco/NotOnco classification accuracy: confusion matrix on the test set.

		Actual Class	
		**Onco**	**NotOnco**
**Prediction Outcome**	**Onco**	152	52
	**NotOnco**	80	180

**Table 2:** Cross-validation accuracy over 10 repetitions for each network configuration setting.

Name	Conv	Pool	Drop	Cross-Validation Accuracy	Standard Deviation
Conf_1	3	2	0.1	68.44%	1.64
Conf_2	3	2	0.3	67.71%	1.92
Conf_3	3	2	0.5	69.49%	2.59
Conf_4	5	2	0.1	68.77%	1.07
Conf_5	5	2	0.3	69.33%	1,78
Conf_6	5	2	0.5	68.82%	1.71
Conf_7	5	3	0.1	69.47%	1.68
Conf_8	5	3	0.3	70.76%	2.40
Conf_9	5	3	0.5	69.63%	2.11
Conf_10	10	5	0.1	70.28%	1.82
Conf_11	10	5	0.3	70.09%	1.55
Conf_12	10	5	0.5	70.44%	2.32

## Abbreviations

The following abbreviations are used in this manuscript:

NGS	Next Generation Sequencing
FISH	Fluorescence In Situ Hybridization
CGH	Comparative Genomic Hybridization
CNN	Convolutional Neural Network
Onco	Class of Oncogenic Sequences
NotOnco	Class of Non Oncogenic Sequences
CDS	Coding Sequence
